# Casein Gene Cluster in Camelids: Comparative Genome Analysis and New Findings on Haplotype Variability and Physical Mapping

**DOI:** 10.3389/fgene.2019.00748

**Published:** 2019-08-29

**Authors:** Alfredo Pauciullo, El Tahir Shuiep, Moses Danlami Ogah, Gianfranco Cosenza, Liliana Di Stasio, Georg Erhardt

**Affiliations:** ^1^Department of Agricultural, Forest and Food Sciences, University of Torino, Grugliasco, Italy; ^2^Institute of Molecular Biology, University of Nyala, Nyala, Sudan; ^3^Department of Animal Science, Nasarawa State University, Keffi, Shabu-Lafia, Nigeria; ^4^Department of Agriculture, University of Napoli Federico II, Portici Italy; ^5^Department for Animal Breeding and Genetics, Justus Liebig University, Gießen, Germany

**Keywords:** camels, casein, haplotype, caseins genes mapping, interspersed element, microsatelite

## Abstract

The structure of casein genes has been fully understood in llamas, whereas in other camelids, this information is still incomplete. In fact, structure and polymorphisms have been identified in three (*CSN1S1*, αs1-CN; *CSN2*, β-CN; *CSN3*, κ-CN) out of four casein genes, whereas controversial information is available for the *CSN1S2* (αs2-CN) in terms of structure and genetic diversity. Data from the genome analysis, whose assembly is available for feral camel, Bactrian, dromedary, and alpaca, can contribute to a better knowledge. However, a majority of the scaffolds available in GenBank are still unplaced, and the comparative annotation is often inaccurate or lacking.Therefore, the aims of this study are 1) to perform a comparative genome analysis and synthesize the literature data on camelids casein cluster; 2) to analyze the casein variability in two dromedary populations (Sudanese and Nigerian) using polymorphisms at *CSN1S1* (c.150G > T), *CSN2* (g.2126A > G), and *CSN3* (g.1029T > C); and 3) to physically map the casein cluster in alpaca. Exon structures, gene and intergenic distances, large insertion/deletion events, SNPs, and microsatellites were annotated. In all camelids, the *CSN1S2* consists of 17 exons, confirming the structure of llama *CSN1S2* gene. The comparative analysis of the complete casein cluster (∼190kb) shows 12,818 polymorphisms. The most polymorphic gene is the *CSN1S1* (99 SNPs in Bactrian *vs*. 248 in dromedary *vs*. 626 in alpaca). The less polymorphic is the *CSN3* in the Bactrian (22 SNPs) and alpaca (301 SNPs), whereas it is the *CSN1S2* in dromedary (79 SNPs). In the two investigated dromedary populations, the allele frequencies for the three markers are slightly different: the allele C at *CSN1S1* is very rare in Nigerian (0.054) and Sudanese dromedaries (0.094), whereas the frequency of the allele G at *CSN2* is almost inverted. Haplotype analysis evidenced GAC as the most frequent (0.288) and TGC as the rarest (0.005). The analysis of R-banding metaphases hybridized with specific probes mapped the casein genes on chromosome 2q21 in alpaca. These data deepen the information on the structure of the casein cluster in camelids and add knowledge on the cytogenetic map and haplotype variability.

## Introduction

Camelids are the only living animals naturally spread over three continents: Africa (dromedaries), Asia (dromedaries and both wild and domesticated Bactrians), and South America (llamas, alpacas, vicunas, and guanacos). Camelids are popular also in Australia (mainly dromedaries) and Europe (llamas and alpacas). However, these populations are not indigenous, but imported from middle of 1800 in Australia (McKnight, 1969), and more recently in Europe. Since their domestication, Old and New world camelids have been exploited as multi-purpose animals for transportation (as beasts of burden), food (as source of milk and meat), but also kept for their fiber (wool and hair), and finally for entertainment (as riding animals). Therefore, these animals are of major economic and cultural importance for nomadic societies of Africa and Asia, as well as for the rural populations of South America.

Although their potential to survive on marginal resources in harsh environment, camelids have not been exploited as an important food source, and in particular of milk. For instance, only 10% of the total milk produced in the rural regions is of camel origin (Faye and Konuspayeva, 2012). Conversely, in the countries of the Gulf, intensive dromedary camel milk production in high-scale modernized unit has been already realized (Faye et al., 2002) and genetic improvement programs for the milk production have been implemented (Nagy et al., 2012).

The daily milk production of dromedary camels is estimated to vary between 3 and 10 kg during a lactation period of 12–18 months (Farah et al., 2007), depending on breed, stage of lactation, feeding, and management conditions, with an average content of 2.9% and 3.1% of protein and fat, respectively (Konuspayeva et al., 2009; Al hay and Al Kanhal, 2010). Data on daily milk production in Bactrians are more variable, depending also by the amount sucked by the calf. On average, it varies between 0.25 and 20 kg per day, with 3.9% of proteins and 5.3% of fat on average (for a review, see Zhao et al., 2015). Conversely, much lower yields were recorded in llamas, whose production ranges between 16 and 413 ml/day during a lactation period reaching a maximum of 220 days (Morin and Rowan, 1995) with an average content of 4.2% and 4.7% of protein and fat, respectively (Riek and Gerken, 2006). In alpacas, milk yield was assessed in a range from 0.4 to 1.2 L/day (Leyva and Markas, 1991).

As for ruminants, the main constituent of camel milk proteins are caseins. Caseins are coded by single autosomal genes, in order *CSN1S1* (αs1-casein), *CSN2* (β-casein), *CSN1S2* (αs2-casein), and *CSN3* (κ-casein), organized as a cluster in a DNA stretch of about 250 kb mapped on chromosome 6 in cattle, sheep, and goat (Rijnkels, 2002). Caseins have been recognized as a powerful molecular model for evolutionary studies (Kawasaki et al., 2011), and their genetic characterization in less investigated species is a useful tool for a better understanding of phylogenetic relationships among domesticated mammalian species and breeds.

In dromedary camels, *CSN2* and *CSN3* genes have been fully characterized (Pauciullo et al., 2013a; Pauciullo et al., 2014), whereas a partial genomic DNA sequence for *CSN1S1* was reported by Shuiep et al. (2013). The casein gene cluster has been investigated also in llama at mRNA level (Pauciullo and Erhardt, 2015) and protein level (Saadaoui et al., 2014), whereas only partial information is known for alpaca (Erhardt et al., 2017).

In dromedary camels, genetic polymorphisms have been identified in three out of four casein genes. Kappeler et al. (1998) described the first two genetic variants (A and B) of *CSN1S1*, which differ for eight amino acids (EQAYFHLE), skipped in A variant as consequence of the alternative splicing of the exon 18 (Erhardt et al., 2016). The C variant was identified at protein level by isoelectrofocusing (IEF) and confirmed at DNA level as polymorphism at the exon 5 (c.150G > T) responsible for the amino acid replacement p.30Glu > Asp (Shuiep et al., 2013). Recently, another variant (D) has been identified by IEF (Erhardt et al., 2016). Apparently, the sequence coding for this variant does not differ from that of the A allele, apart from an insertion of 11 bp in the intron 17, which may affect the spliceosome machinery then generating the skipping of the exon 18 (Erhardt et al., 2016). Genetic variants have been described also for the *CSN2* and *CSN3*. The SNP g.2126A > G at *CSN2* and g.1029T > C at *CSN3* are particularly relevant for changing consensus sequences for transcription factors (TATA-box and HNF-1, respectively) (Pauciullo et al., 2013a; Pauciullo et al., 2014). Conversely, controversial information on exons’ number is available for the *CSN1S2* gene, and no SNP has been reported so far for the αs2-casein, despite a series of alternative splicing variants have been recently described by Ryskaliyeva et al. (2019). However, in this respect, useful data may derive from the genome analysis, whose assembly is available on line for feral, Bactrian, and dromedary camel, as well as for alpaca. The complete sequence is made of about 2,000 Mbases each species, but the isolated genomic scaffolds available in GenBank are still unplaced, and their annotation is almost completely lacking (Avila et al., 2014a). This observation underlines the need to acquire more data to help the annotation of the camel genome. Furthermore, considering the tight association among the casein genes, the estimation of the relationship between casein variants and milk production traits can be improved by considering the casein haplotypes instead of single genes.

The karyotype structure of camelids (2n = 74) and their similarities have been elucidated (Bunch et al., 1985; Di Berardino et al., 2006). However, lack of information exists in the cytogenetic mapping of genes, being located only few hundreds (Avila et al., 2014b; Perelman et al., 2018) not including casein loci that are important for their link with favorable/undesirable characteristic of coat color fibers, as observed in other species (Grosz and MacNeil, 1999).

Therefore, aims of the of this study are 1) to propose a revised and detailed comparative analysis of the casein cluster in Bactrian, dromedary, and alpacas using the feral camel genome as reference and the annotation available for all casein transcripts in llama; 2) to analyze the casein cluster variability in two dromedary populations (Sudanese and Nigerian) using genetic markers at *CSN1S1*, *CSN2*, and *CSN3*; and 3) to physically map the casein genes in alpaca.

## Materials and Methods

In order to accomplish the aims of the study, a dual approach was used. A multiple bioinformatics analysis of the genomes was achieved to elucidate the cluster and gene organization, the level of genetic diversity (SNP and microsatellites), the variability in the Interspersed elements, and the type of regulatory elements of the gene expression. A laboratory approach was accomplished to genotype and establish haplotypes in the dromedaries and to map cytogenetically the genes in alpaca.

### Genome Comparative Analysis

The contig 039344 available in EMBL with the acc. no. AGVR01039100.1 and isolated from the whole genome sequence of the feral camel (Wang et al., 2012) was used as reference to establish sizes, positions, and orientations of the genes belonging to casein cluster. Scaffolds 146 (NW_011517196), 313 (NW_011591251), and 223 (KN269544) belonging respectively to Bactrian, dromedary, and alpaca genomes were used in the comparative analysis to describe differences in the casein cluster and to detect inter-specific genetic diversity.

Homology searches, comparison among sequences, and multiple alignments were achieved using MEGA 4 software (Tamura et al., 2007), whereas repeat masking was performed by Censor software (Kohany et al., 2006). Microsatellites were found by BioPHP Microsatellite repeat finder (http://insilico.ehu.es/mini_tools/microsatellites/). The main putative transcription factor binding sites were searched by TFBIND software considering 85% as minimum binding score.

Computational analysis of spliceosome specific sites was achieved by FruitFly software (http://www.fruitfly.org/seq_tools/splice.html), whereas the protein secondary structure was predicted by Jpred 4 software (http://www.compbio.dundee.ac.uk/jpred/), and the impact on protein biological functions was assessed by PROVEAN (Protein Variation Effect Analyser) software (http://provean.jcvi.org/index.php).

Genepop software was used to estimate allele frequencies and to test for Hardy-Weinberg equilibrium (뇸χ2 test). Casein haplotype frequencies were estimated by PHASE ver.2.1 (Li and Stephens, 2003).

### Ethics Approval Statement

Samples collection from dromedary followed all institutional and specific national guidelines for the care and use of laboratory animals. In particular, protocols were approved by Research and Ethics Committees of the Nasarawa State University (approval no: NSU/REC/AGRO10) for Nigerian camels and authorized by Ministry of Animal Resources and Fisheries (no number is available) for Sudanese dromedaries.

The collection of alpaca samples was done according to the German Animal Welfare Act. On the basis of article 8 (7) 2a of this law, no notification of or approval by the Animal Protection Unit of the Regional Council of Giessen, Germany, was necessary for this study.

### *Camelus dromedarius* DNA Samples

A total of 267 blood samples were collected from dromedaries in Sudan and Nigeria. Samples were considered as representative of both countries because they were collected in different regions. Those from Sudan came from five areas: El Shuak (El Gadarif State), West Omdurman (Khartoum State), El Obeid (North Kordofan State), Nyala (South Darfur State), and Tamboul (El Butana area). Those from Nigeria came from Kano and Sokoto areas (North and North-west regions, respectively).

In particular, 198 Sudanese she-camels belonging to different ecotypes including Shanbali, Kahli, Lahaoi, and Arabi dromedary camels were provided by University of Nyala (Nyala, South Darfur, Sudan) and collected between years 2011 and 2012, whereas 69 Nigerian autochthonous dromedary camels were provided by Nasarawa State University (Nigeria) and collected between years 2016 and 2017.

DNA was isolated from blood leucocytes with the procedure already described by Sambrook et al. (1989).

DNA concentration and OD_260/280_ ratio were measured with the Nanodrop ND-1000 Spectrophotometer (Thermo Fisher Scientific Inc., Waltham, MA, USA).

#### Genotyping at Dromedary Camel *CSN1S1*, *CSN2*, and *CSN3* by PCR-RFLP Methods

Genotyping was carried out at DNA level using the methods described by Shuiep et al. (2013) for the c.150G > T at *CSN1S1* (allele C), Pauciullo et al. (2014) for the g.2126A > G at *CSN2*, and Pauciullo et al. (2013a) for the g.1029T > C at *CSN3*. Primer sequences, the thermal amplification conditions, and the list of restriction enzymes are reported in **Table 1**. PCR amplification was carried out using Bio-Rad T100 thermocycler (Bio-Rad). The digestion products were analyzed directly by electrophoresis in 2.5% agarose gel in 1X TBE buffer and stained with ethidium bromide.

**Table 1:** Sequences and annealing temperature of the primers used for the genotyping by PCR-RFLP assays **(A)** and for preparation of the FISH probes covering the casein genes cluster **(B)**. All primers were designed on wild feral camel genome sequence available in gene bank (EMBL acc. no. AGVR01039100.1), and multiple alignment confirmed 100% similarity in the other camelids.

SNP (A)	GenBank ID	Primers		Annealing temperature (°C)	Size (bp)	Genotyping
*CSN1S1*	JF429138	Forward:	5’-TGAACCAGACAGCATAGAG-3’	58.5	930	*SmlI*
c.150G > T		Reverse:	5’-CTAAACTGAATGGGTGAAAC-3’			
*CSN2*	HG969421	Forward:	5’-GTTTCTCCATTACAGCATC-3’	60.0	659	*HphI*
g.2126A > G		Reverse:	5’-TCAAATCTATACAGGCACTT-3’			
*CSN3*	HE863813	Forward:	5’-CACAAAGATGACTCTGCTATCG-3’	62.0	488	*AluI*
g.1029T > C		Reverse:	5’-GCCCTCCACATATGTCTG-3’			
Probe (B)	Gene	Primers		Annealing temperature (°C)	Size (bp)	Position
1	*CSN1S1*	Forward	5’-GTACCCAGAAGTCTTTCAA-3’	59.5	913	Exon 3
		Reverse	5’-CACTGCTAACTCAAGAATCT-3’			Exon 5
2	*CSN2*	Forward	5’-TTCACTTCTTTTCCTCCAC-3’	62.3	2433	Exon 1
		Reverse	5’-CCATTGTATTTGTGCAATATTA-3’			Intron 1
3	*CSN2*	Forward	5’-GATGAACAGCAGGATAAAATC-3’	56.0	657	Exon 7
		Reverse	5’-ATCACTGATCTGAACTAT-3’			Intron 7
4	*CSN1S2*	Forward	5’-AGCTGTAAGGAACATAAAGG-3’	60.5	1493	Exon 7
		Reverse	5’-TGTGGGGACTTCAGCTG-3’			Exon 8
5	*CSN3*	Forward	5’-TGCAGAGGTGCAAAACCA -3’	61.5	1337	Exon 4
		Reverse	5’-GCTAGTCTGTGTTGGTAGTAA-3’			Exon 5

#### Karyotyping and Probe Preparation

Peripheral blood cell cultures from two German alpacas were treated for late incorporation of BrdU (15 mg/ml) to obtain R-banding preparations. Hoechst 33258 (30 mg/ml) was simultaneously added to BrdU 6 h before harvesting to enhance the R-banding patterns. The alpacas were karyotyped according to standard methods for RBA-banding techniques (Iannuzzi and Di Berardino, 2008). Chromosome identification followed the R-banded ideogram of *Vicugna pacos* (2n = 74) chromosomes (Di Berardino et al., 2006). The R-banding preparations were further used for FISH analysis.

The casein gene probes were prepared by PCR amplification and cloning of five DNA fragments spread over the casein genes (primers are provided in **Table 1**) according to the method described by Pauciullo et al. (2013b). Labeling was carried out by standard nick translation reactions (Roche, Germany) using biotin-16-dUTP (Roche) as modified nucleotide. The probes were then used for FISH analysis.

#### Fluorescent *In Situ* Hybridization (FISH)

RPBI-FISH was performed according to Pauciullo et al. (2013b) and Pauciullo et al. (2016) with minor modifications. Briefly, 500 ng of labeled DNA from each of the nick translation reactions were combined and mixed together with competitor DNA. The probes were precipitated in ethanol 100% and then reconstituted in 7 µl hybridization solution (50% formamide in 2X SSC + 10% dextran sulfate), denatured at 75°C for 10 min, and incubated at 37°C for 60 min for pre-hybridization.

Fixed R-banding metaphase plates were stained with Hoechst 33258 (25 µg/ml) for 10 min, then washed, mounted in 2X SSC (pH 7.0), and exposed to UV light for 30 min to reinforce the banding. The slides were then denatured for 3 min in a solution of 70% formamide in 2X SSC (pH 7.0) at 75°C.

The hybridization mixture was applied to the slides and incubated in a moist chamber at 37°C for 3 days. Detection was performed three times with 1:400 fluorescein isothiocyanate (FITC)-avidin (Vector Laboratories, CA, USA) and 1:200 anti-avidin antibody (Vector Laboratories, CA, USA). Finally, slides were mounted with antifade/propidium iodide (3 µg/ml) and observed at 100× magnification with a Leica DM5500 fluorescence microscope equipped with FITC and Texas Red (TXRD) specific filters and provided with a CytoVision MB 8 image-analysis system (Leica Microsystems, Wetzlar, Germany).

A total of 30 randomly selected metaphase cells were examined per each alpaca to ensure the reliability of the probe signals by FISH. The hybridization efficiency was calculated as follows: FISH efficiency (%) is equal to the number of cells with hybridization signals present at the 2q21 region of both chromosomes 2 divided by the number of cells examined (Pauciullo et al., 2013b).

## Results

### Multiple Bioinformatics Analysis

#### Cluster Organization

**Figure 1:**
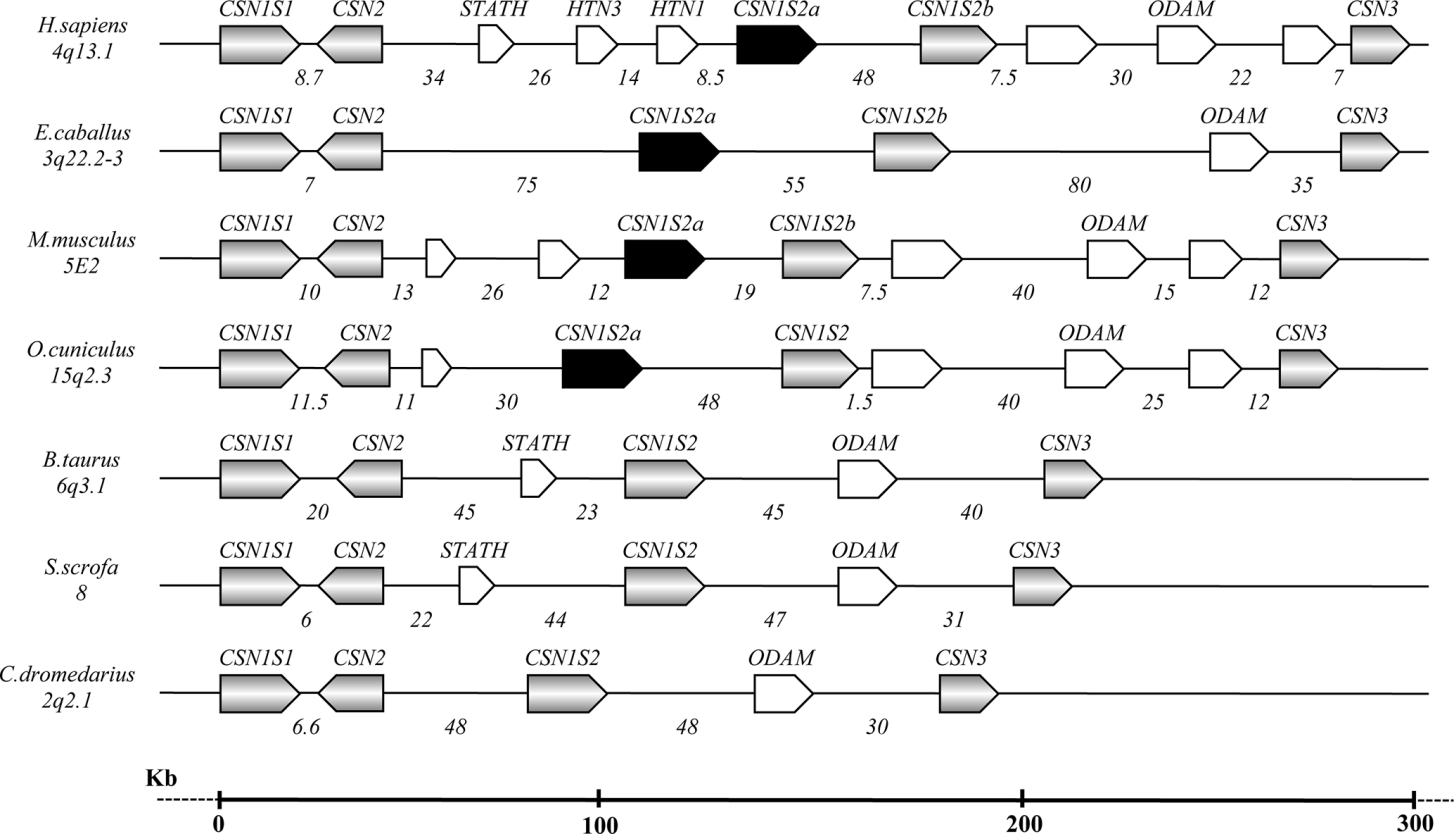
Schematic representation of the casein gene cluster in different species, chromosome location, gene orientation, and related intergenic distances based on comparative genomic analysis (Modified from Martin et al., 2013 and Ryskaliyeva et al., 2018).

The caseins of camelids are encoded by four genes tightly clustered in a DNA fragment of about 190 kb. The organization and the orientation of the genes are highly conserved compared to all species studied to date, although with large differences in sizes partially due to a diverse number and natures of the interspersed repeated elements [short interspersed elements (SINEs), long interspersed elements (LINEs), microRNA (miR), etc.], partially due to genome expansion events and a higher number of genes present (**Figure 1**).

The first two casein genes (*CSN1S1* and *CSN2*) are close up (6.6 kb) compared to higher intergenic distances of the other casein genes (**Table 2**). For instance, a large distance (85.7 kb) exists between the *CSN1S2* and *CSN3*. In this interval, the *ODAM* gene was found, whereas no other known genes were found in the intergenic intervals *CSN1S1-CSN2* and *CSN2-CSN1S2*.

**Table 2:** Positions, sizes, and exon numbers of the casein genes and related intergenic distances occurring in the cluster. The contig 039344 available in EMBL with the acc. no. AGVR01039100.1 and isolated from the whole genome sequence of the feral camel (Bactrian Camels Genome Sequencing and Analysis Consortium) was used as reference. D, Dromedary; C, other camelids; L, Llama (Pauciullo and Erhardt, 2015).

Gene	Position	Size (bp)^A^	Intergenic distance (bp)^B^	Total size (bp)^A+B^	Exons
*CSN1S1*	242,112 to 258,587	16,476			20^D^/21^C^
↓			6,600		
*CSN2*	265,187 to 273,094	7,908			9
↓			48,261		
*CSN1S2*	321,355 to 335,898	14,544			17
↓			85,699		
*CSN3*	421,597 to 430,955	9,359			5^D,C^/6^L^
				188,847	

The comparative analysis of the genome sequence of wild feral camel (EMBL acc. no. AGVR01039100.1) with the annotated casein genes in dromedaries (HG969421; HE863813) and cDNAs of the whole cluster in llama (EMBL acc. nos. LK999986; LK999992; LK999989; LK999995) allowed the complete exon identification in all camelids. The *CSN1S1* is made of 20 exons in dromedary and 21 exons in the other camelids, the *CSN2* consisted of nine exons, the *CSN1S2* is arranged in 17 exons, and the *CSN3* is organized in five exons (**Table 2**). Splice donor and acceptor consensus sequences conforming to the 5’-GT/3’-AG rule were identified at the exon/intron boundaries. The average GC content of the complete DNA interval (*CSN1S1* to *CSN3*) is 34%, and the average repeat content is about 20% (**Supplementary Table 1**).

#### Single Nucleotide Polymorphisms and Microsatellites

The comparative analysis of the complete casein cluster (∼190kb) showed a total of 12,818 SNPs (**Table 3**). For all camelids, the most polymorphic gene was the *CSN1S1* (99 SNPs in Bactrian *vs*. 248 in dromedary *vs*. 626 in alpaca), whereas the less polymorphic gene was the *CSN3* in Bactrian (22 SNPs) and alpaca (301 SNPs) and the *CSN1S2* in dromedary (79 SNPs).

**Table 3:** Number of polymorphic sites differentiated in substitutions (Sub), insertions (Ins), and deletions (Del) found in the casein cluster region of Bactrian, dromedary, and alpaca by the comparative genomic analysis using the wild camel sequence (AGVR01039100.1) as reference, including the total numbers of polymorphic sites (TOT) within gene by species and in total.

	C. bactrianus				C. dromedarius				V. pacos				TOT
	Sub	Ins	Del	TOT	Sub	Ins	Del	TOT	Sub	Ins	Del	TOT	
***CSN1S1***	17	16	66	**99**	50	141	57	**248**	333	248	45	**626**	**973**
Intergenic 1	6	0	0	**6**	23	3	0	**26**	206	15	23	**244**	**276**
***CSN2***	7	0	90	**97**	36	87	91	**214**	216	8	141	**365**	**676**
Intergenic 2	70	32	379	**481**	216	156	378	**750**	1085	544	825	**2,454**	**3,685**
***CSN1S2***	24	15	17	**56**	41	28	10	**79**	360	74	75	**509**	**644**
Intergenic 3	56	27	105	**188**	234	84	99	**417**	1406	419	342	**2,167**	**2,772**
ODAM	5	1	2	**8**	30	13	4	**47**	172	17	36	**225**	**280**
Intergenic 4	92	165	51	**308**	148	138	142	**428**	722	80	1405	**2,207**	**2,943**
***CSN3***	15	4	3	**22**	38	65	143	**246**	220	16	65	**301**	**569**
**TOT**	**292**	**260**	**713**	**1,265**	**816**	**715**	**924**	**2,455**	**4,720**	**1,421**	**2,957**	**9,098**	**12,818**

The analysis of sequences for microsatellites discovery found a total of 35 microsatellites. Fifteen were identified in all camelids, six were shared among three species, seven were in common between two species, whereas seven were found to be species-specific (**Table 4**). No specific microsatellites were found in the wild feral camel.

**Table 4:** List of microsatellites found in the casein cluster of camelids. Short tandem repeats polymorphic among the species are in italics. Species-specific microsatellites correspond to gray cells. Positions are indicated according to the corresponding GenBank sequence (wild feral: AGVR01039100.1; Bactrian: NW_011517196.1; dromedary: NW_011591251; alpaca: KN269544); therefore, they are complementary (Compl) for Bactrian and dromedary.

Wild feral				Bactrian				Dromedary				Alpaca			
Position	Cicle	Repeats	Unit	Position Compl	Cicle	Repeats	Unit	Position Compl	Cicle	Repeats	Unit	Position	Cicle	Repeats	Unit
254698	2	7	TC	7090479	2	7	TC	491968	2	7	TC	353736	2	7	TC
*254760*	*2*	*19*	*TG*	*7090418*	*2*	*27*	*TG*	*491907*	*2*	*26*	*TG*	*353797*	*2*	*17*	*TG*
257864	2	6	TG									356896	2	6	TG
*274229*	*2*	*8*	*TA*	*7071025*	*2*	*10*	*TA*	*472362*	*2*	*8*	*TA*	*373152*	*2*	*7*	*TA*
				7069900	3	10	TA								
*275356*	*3*	*7*	*TAT*					*471241*	3	6	TAT				
293407	2	6	TA	7051865	2	6	TA	453324	2	6	TA	391869	2	6	TA
*294330*	*3*	*9*	*TAT*									*392786*	*3*	*15*	*TAT*
*294361*	*3*	*9*	*ATC*	*7050923*	*3*	*10*	*ATC*	*452355*	*3*	*9*	*ATC*	*392832*	*3*	*10*	*ATC*
								441188	4	6	ATTG				
*327761*	*4*	*7*	*AGAC*	*7017880*	*4*	*7*	*AGAC*	*419012*	*4*	*10*	*AGAC*				
												446456	3	10	TCC
348071	3	7	CCG	6997586	3	7	CCG					446668	3	7	CCG
348583	2	6	TC	6997084	2	6	TC	398200	2	6	TC				
*349673*	*2*	*9*	*TG*	*6995994*	*2*	*11*	*TG*	*397110*	*2*	*7*	*TG*	*448262*	*2*	*6*	*TG*
*349701*	*2*	*6*	*TA*	*6995962*	*2*	*6*	*TA*	*397086*	*2*	*6*	*TA*	*448282*	*2*	*10*	*TA*
*362062*	*4*	*10*	*TAGA*	*6983602*	*4*	*9*	*TAGA*	*384718*	*4*	*12*	*TAGA*				
362796	2	6	AT	6982856	2	6	AT	383956	2	6	AT	461613	2	6	AT
												461638	2	6	TA
369500	2	7	CA	6976226	2	7	CA	377270	2	7	CA	468360	2	7	CA
373342	2	9	TC	6972383	2	9	TC								
*373360*	*2*	*12*	*AC*	*6972365*	*2*	*16*	*AC*	*373405*	*2*	*14*	*CA*	*472211*	*2*	*7*	*CA*
												488423	2	6	AC
												495913	2	6	TA
397130	2	6	AT	6948637	2	6	AT	349656	2	6	AT	495926	2	7	AT
				6943100	4	10	TAAC	344115	4	9	TAAC				
*404226*	*2*	*7*	*CA*	*6941532*	*2*	*7*	*CA*	*342508*	*2*	*11*	*CA*	*502998*	*2*	*15*	*CA*
*406619*	*2*	*12*	*TG*	*6939139*	*2*	*12*	*TG*	*340113*	*2*	*14*	*TG*	*505398*	*2*	*8*	*TG*
*408958*	*3*	*8*	*ATG*	*6936762*	*3*	*11*	*ATG*	*337850*	*3*	*8*	*ATG*	*507654*	*3*	*6*	*ATG*
*412396*	*2*	*8*	*AC*	*6933319*	*2*	*13*	*AC*	*334433*	*2*	*11*	*AC*	*511052*	*2*	*16*	*AC*
415959	2	6	TA	6929747	2	6	TA	330866	2	6	TA				
								330472	2	8	TG				
*416355*	*2*	*10*	*TG*	*6929351*	*2*	*10*	*TG*	*330404*	*2*	*7*	*TG*				
416377	2	6	CA	6929329	2	6	CA								
				*6914165*	*2*	*11*	*AC*	*315383*	*2*	*10*	*AC*				

#### Interspersed Elements

**Figure 2:**
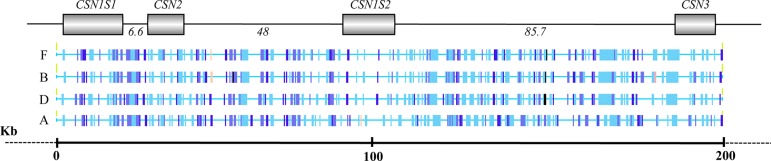
Overview of the interspersed elements identified in the casein gene cluster in wild feral camel **(F)**, Bactrian **(B)**, dromedary **(D)**, and alpaca **(A)**.

The analysis of the interspersed repeats evidenced a total of 696 elements (169 in wild camel, 171 in Bactrian, 174 in dromedary, and 182 in alpaca). Most of them (82.3%; 572 elements) were common among all camelids; 84 interspersed repeats (12.0%) were shared between two (18 elements) or three species (66 elements), whereas 40 interspersed elements (5.7%) were species-specific (**Supplementary Table 1**). The diagrammatic representation of interspersed elements is reported in **Figure 2**.

Alpaca showed 27 species specific transpositions, whereas the Old word camelids were poor in these elements, only 12 in total. In particular, four interspersed elements, mainly belonging to LINE-1 (L1) retrotransposons, were found in the wild feral; three repetitive sequences (MER35, ERV_36_MD_I, RLTR25_MM) were detected in the Bactrian; and five specific repetitive elements (CHARLIE8; miR; LTR2_Vpa; SloEFV-I; L1MAB2_ML) were found in dromedary (**Table 5**).

**Table 5:** Species-specific interspersed elements found by the comparative genomic analysis of the casein cluster in camelids (A, alpaca; B, Bactrian; D, dromedary; F, feral camel) and listed in order 5’ > 3’ as they appear in the cluster. Repbase was used as repeat database. Positions are indicated according to the corresponding GenBank sequence (wild feral: AGVR01039100.1; Bactrian: NW_011517196.1; dromedary: NW_011591251; alpaca: KN269544). The direction of the repeat fragment is indicated as d = direct or c = complementary; Sim, similarity with repeat element in the database; and Pos/Mm, Ts column is a ratio of mismatches to transitions in nucleotide alignments. The closer Pos/Mm, Ts number is to 1 the more likely is that mutations are evolutionary.

Species		Position	Name	Class	Dir	Sim	Pos/Mm : Ts	Size (bp)
A		345745-345817	ERV3-1_SSc-I	ERV	c	0.785	2.00	72
		352418-352502	ERV44_MD_I	ERV	c	0.712	1.47	84
D	Compl. to	485496-485356	CHARLIE8	DNA/hAT	c	0.647	1.59	140
	Compl. to	478096-478030	miR	SINE	c	0.806	1.22	66
F		268407-268493	THER2	SINE	c	0.755	2.00	86
A		369205-369268	MER4BI	ERV	c	0.822	2.00	63
		372430-372493	L1-2B_EC	LINE	d	0.790	1.50	63
		381127-381183	MARINER4_MD	Mariner	d	0.836	1.00	56
B	Compl. to	7053559-7053474	MER35	MER	d	0.717	1.58	85
A		391846-391888	L1A-2_MD	LINE	d	0.809	1.17	42
		395574-395643	Zaphod3	DNA/hAT	d	0.760	1.18	69
		408816-408940	ERV3-5-EC_LTR	ERV	c	0.830	2.11	124
F		310230-310295	L1-2_Vpa	LINE	d	0.750	1.67	65
A		413499-413572	HAL1-3_ML	LINE	c	0.783	1.50	73
		417674-417832	L2	CR1	d	0.652	1.47	158
		417888-418114	L2	CR1	d	0.654	1.83	226
D	Compl. to	426349-425998	LTR2_Vpa	ERV	d	0.758	4.61	351
A		422684-422764	LTR28_OC	ERV	c	0.707	1.62	80
		426397-426425	SQR2_MM	Sat	d	0.931	2.00	28
		427755-427846	L1-2_Vpa	LINE	d	0.768	1.80	91
		451056-451088	HERVK3I	ERV	d	0.882	1.50	32
D	Compl. to	383725-383659	SloEFV-I	ERV	d	0.776	1.83	66
A		465703-465770	MER104B	DNA	c	0.753	1.78	67
		468337-468372	ERV2-1-I_Opr	ERV	c	0.865	1.00	35
		471920-471998	RMER3D-int	ERV	d	0.782	1.83	78
		478503-478578	LTR16	ERV	c	0.701	1.69	75
		480865-480899	SINE_VV	SINE	c	0.888	1.50	34
F		393221-393292	L1-1H_Cpo	LINE	d	0.833	2.00	71
A		493574-493639	MER28	Mariner	d	0.791	1.44	65
		502950-503003	RLTR17B_Mm	ERV	c	0.763	1.33	53
		505352-505434	RLTR17_MM	ERV	d	0.765	1.56	82
B	Compl. to	6936860-6936781	ERV36_MD_I	ERV	d	0.779	1.71	79
A		509996-510114	PRIMA4_I	ERV	d	0.736	1.85	118
B	Compl. to	6933341-6933305	RLTR25_MM	LTR	c	0.8611	2.00	36
F		412370-412416	L1-3_TS	LINE	c	0.847	9.90	46
A		519191-519256	L1MdV_II	LINE	c	0.761	1.44	65
D	Compl. to	328118-328009	L1MAB2_ML	LINE	d	0.684	1.48	109
A		519318-519379	ERV2-3_STr-I	ERV	d	0.796	1.50	61
		524222-524291	UCON28c	Int. Rep.	c	0.843	2.50	69

#### Promoters

The analysis of the casein gene promoters for the discovery of the consensus sequences for transcription factors evidenced 505 binding sites with a score ranging between 85% and 100%. The most representative elements correlated to protein and milk production were those belonging to the Oct family (octamer-binding elements), GATA-binding proteins, CCAAT-enhancer-binding proteins (C/EBPs), and broad activators like AP-1, AP-2, SP1, etc. The consensus sequences common to the four caseins and showing the highest binding scores are reported in **Table 6**.

**Table 6:** Most representative consensus motifs for transcription factors detected in the 5’-flanking regions of camelids by TFBIND software and present in all caseins with higher binding score (BS). DNA strands (S) in direction 5’ > 3’ are indicated by +. The opposite strands are indicated by -.

Transcription factor	Consensus motif	*CSN1S1*			*CSN2*			*CSN1S2*			*CSN3*		
		Position	S	BS	Position	S	BS	Position	S	BS	Position	S	BS
AML1/Runx	TGTGGT	-259/-254	-	0.873	-57/-52	-	1.000	-300/-295	+	0.910	-61/-56	-	0.850
AP-1	RSTGACTNMNW	-186/-176	-	0.850				-98/-88	+	0.851	-104/-94	-	0.890
C/EBP	NNTKTGGWNANNN	-304/-292	-	0.940	-271/-259	+	0.911	-51/-39	-	0.927	-58/-45	-	0.875
GATA	NNNGATRNNN	-106/-97	+	0.870	-183/-174	+	0.887	-350/-341	-	0.860	-124/-115	-	0.931
HNF3	NNNTRTTTRYTY	-83/-72	+	0.880	-77/-66	+	0.928	-338/-327	+	0.928	-20/-9	+	0.932
MyoD	SRACAGGTGKYG				-307/-296	+	0.874	-265/-254	+	0.925	-62/-51	-	0.858
Oct-1	NNNRTAATNANNN	-267/-255	-	0.929	-131/-120	+	0.917	-186/-174	+	0.947	-84/-72	+	0.852
Pbx-1	ANCAATCAW	-45/-37	+	0.942	-109/-101	+	0.903	-221/-213	+	0.899	-33/-25	-	0.912
SRY	AAACWAM	-253/-248	+	0.941	-192/-186	-	0.960	-169/-163	+	0.947	-14/-8	-	0.939
MGF/STAT5	TTCCCRKAA	-270/-262	+	0.931	-94/-86	-	0.870	-292/-284	-	0.956			
TATA-box	WTATAAAW	-31/-25	+	0.980	-28/-19	+	0.910	-23/-16	+	0.865	-18/-11	-	0.927

### Experimental Data

#### Genotype and Haplotype Analyses in Dromedaries

**Figure 3:**
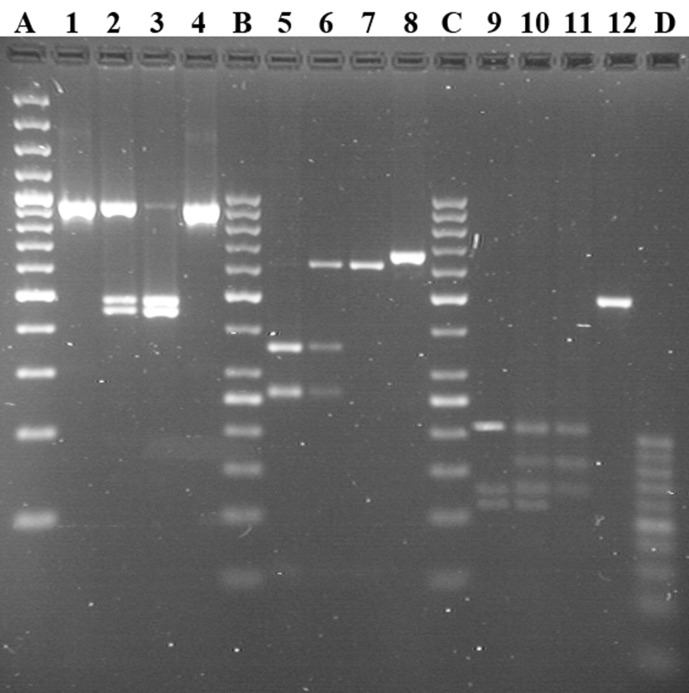
Genotyping of *CSN1S1*, *CSN2*, and *CSN3* by PCR-RFLP in Sudanese and Nigerian dromedary populations. Lines 1–4: locus *CSN1S1* c.150G > T; genotypes TT, GT, and GG reported in lines 1, 2, and 3, respectively. Lines 5–8: locus *CSN2* g.2126A > G; genotypes GG, AG, and AA reported in lines 5, 6, and 7, respectively. Lines 9–12: locus *CSN3* g.1029T > C; genotypes CC, TC, and TT reported in lines 9, 10, and 11, respectively. Lines 4, 8, and 12 show undigested PCR products each belonging to the relative locus. Line A shows the GeneRuler™ 100 bp plus DNA Ladder (Thermo Scientific). Lines B and C show GeneRuler™ 50 bp DNA Ladder (Thermo Scientific). Line D shows 20bp DNA Ladder (Jena Bioscience).

Two dromedary populations (Sudanese and Nigerian) were genotyped for SNPs located on three genes (c.150G > T, *CSN1S1* allele C; g.2126A > G, *CSN2* promoter; and g.1029T > C, *CSN3* promoter), known for being polymorphic. **Figure 3** shows the genotype pattern for the three polymorphisms.

The allelic frequencies are reported in the **Table 7**. The allele C at *CSN1S1* is very rare in Nigerian (0.054) and Sudanese dromedaries (0.094), whereas the frequency of the allele G at *CSN2* is almost inverted (0.550 in Nigerian *vs*. 0.350 in Sudanese), as happens also for the allele C at *CSN3* (0.549 in Nigerian *vs*. 0.377 in Sudanese). No deviation from Hardy-Weinberg equilibrium was found for all loci within populations.

**Table 7:** Allele and haplotype frequencies detected for the SNPs c.150G > T, g.2126A > G, and g.1029T > C at the casein loci in Sudanese and Nigerian dromedary populations.

Allele frequencies								
	*CSN1S1*			*CSN2*			*CSN3*	
	c.150G > T			g.2126A > G			g.1029T > C	
	G	T		A	G		T	C
**Sudanese (n = 198)**	**0.906**	**0.094**		**0.650**	**0.350**		**0.623**	**0.377**
Shanbali	0.900	0.100		0.640	0.360		0.540	0.460
Khali	0.921	0.079		0.723	0.277		0.700	0.300
Arabi	0.942	0.058		0.704	0.296		0.654	0.346
Lahaoi	0.888	0.112		0.587	0.413		0.607	0.393
**Nigerian (n = 69)**	**0.946**	**0.054**		**0.450**	**0.550**		**0.451**	**0.549**
Haplotype frequencies								
	1	2	3	4	5	6	7	8
	**GAC**	**GAT**	**GGC**	**GGT**	**TAC**	**TAT**	**TGC**	**TGT**
**Sudanese**	**0.348**	**0.263**	**0.028**	**0.269**	**0.007**	**0.026**	**0.004**	**0.052**
Shanbali	0.415	0.126	0.003	0.354	0.019	0.067	0.000	0.013
Khali	0.277	0.401	0.039	0.203	0.016	0.037	0.001	0.023
Arabi	0.254	0.374	0.071	0.241	0.024	0.019	0.004	0.009
Lahaoi	0.347	0.239	0.039	0.269	0.001	0.001	0.015	0.087
**Nigerian**	**0.187**	**0.226**	**0.290**	**0.223**	**0.014**	**0.029**	**0.006**	**0.023**
**Over-all frequency**	**0.288**	**0.271**	**0.099**	**0.253**	**0.011**	**0.027**	**0.005**	**0.042**
Standard Error	0.018	0.021	0.015	0.019	0.006	0.007	0.004	0.008

In bold the the allele and haplotype frequencies for the complete Sudanese and Nigerian populations analysed, as well as for the over-all frequency.

On the basis of the genotypes detected for each *locus*, eight haplotypes were observed in both populations (**Table 7**). Sudanese camels showed a higher frequency (0.348) of the haplotype GAC compared to the Nigerian (0.187), where the most represented haplotype (0.290) was GGC, rather underrepresented in the Sudanese camels (0.028). Overall, the haplotype GAC was the most frequent (0.288), whereas TGC was the rarest (0.005).

#### Cytogenetic Mapping

The investigated alpacas were karyotyped, and the analysis of the RBA-banding pattern showed karyologically normal animals (2n = 74, XX).

**Figure 4:**
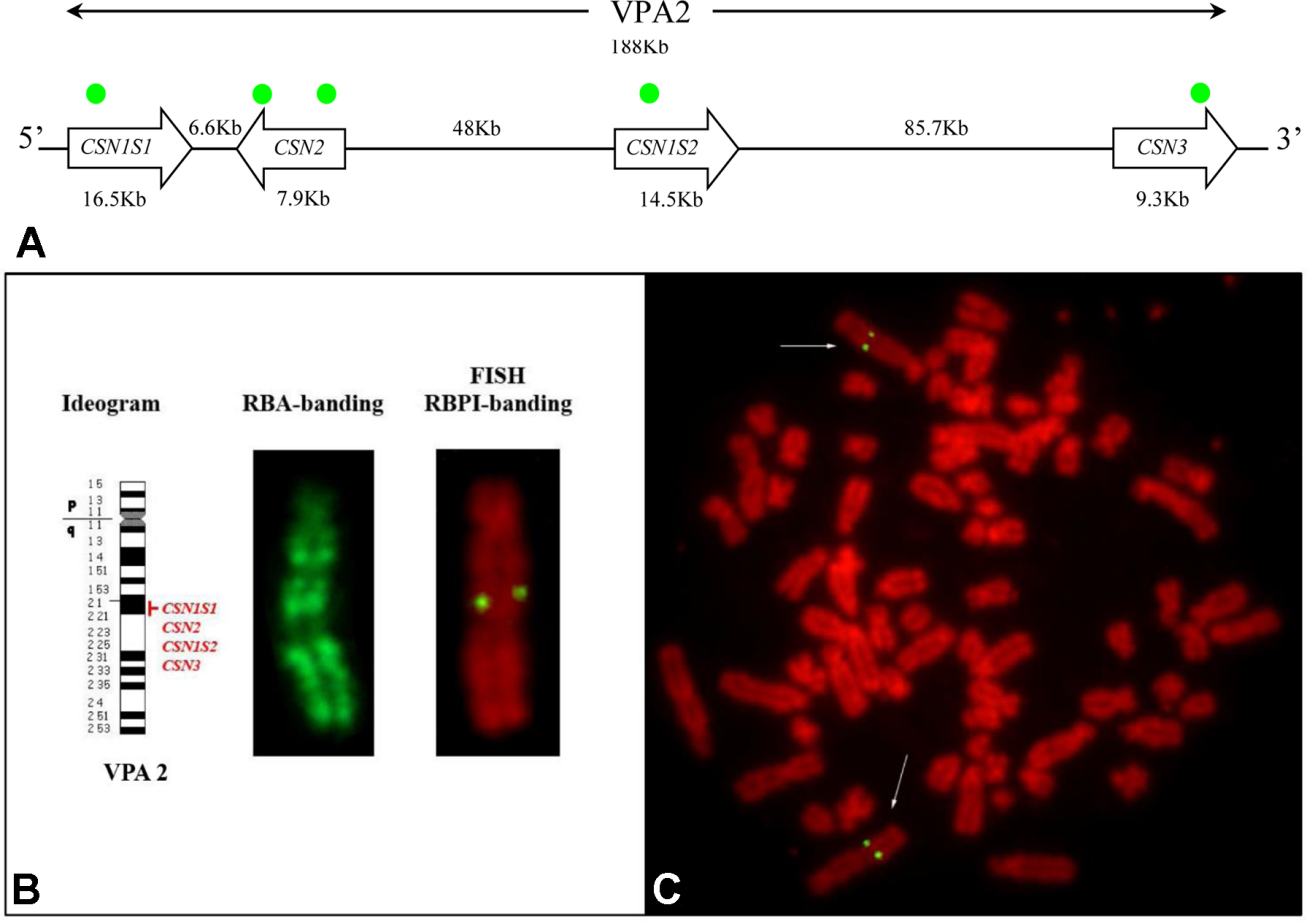
Casein cluster organization and genes mapping in alpaca (VPA). **(A)** Schematic representation of the genomic arrangement of the casein loci along the chromosome. The approximate position of the gene-specific probes used for the mapping is indicated with green dots. **(B) **Diagrammatic representation of VPA 2, RBA-banding, and RBPI staining. (**C**)****Fluorescence *in situ* hybridization obtained by using *CSN1S1*, *CSN2*, *CSN1S2*, and *CSN3* gene probes shows specific signal on chromosome 2q2.1. Fluorescein isothiocyanate signals were superimposed on RBPI-banding (R-banding using early BrdU-incorporation and propidium iodide staining).

Five PCR amplicons spanning the casein *loci* were mixed together and used to set up a fluorescence *in situ* hybridization (FISH) based method for the mapping on alpaca chromosomes (**Figure 4**). The specificity of the amplified probes was first assessed by agarose gel electrophoresis and then by Sanger sequencing. The comparison with the feral camel genome sequence (EMBL acc. no. AGVR01039100.1) and with the homologous camel *CSN2* (EMBL acc. no. HG969421) and *CSN3* (EMBL acc. no. HE863813) gene sequences confirmed that the probes belonged to the casein genes.

The reliability of the gene signal detection by FISH was assessed on 30 counted metaphases. The FISH efficiency was 92.2% on average (range 86–97%). Two couple of symmetrical spots, each belonging to the sister chromatids of the two homologous chromosomes, were identified in the analyzed R-banding metaphases (**Figure 4B, C**). The application of the propidium iodide-staining (RBPI-FISH) allowed the mapping of the casein genes to the chromosome 2q21. Alpaca chromosome 2 is reported in detail in **Figure 4B**.

No further hybridization signals were detected on the other chromosomes, thus confirming the cluster organization of casein genes with no duplications (**Figure 4A**).

## Discussion

The dramatic progress of sequencing technologies and the enormous reduction in the cost of sequencing opened the era of the genomics. Genome sequencing projects provided a huge amount of data, but, despite the new research abilities have been developed, new problems are also coming out. For example, the low coverage assembly and the tentative annotations often built on the human genome, led to repetitive information, exon losses, and errors in gene annotations. This is still more evident for less explored species like those belonging to *Camelidae*. For instance, the *CSN2* in the alpaca genome has been annotated without the exon 3, because this exon is out-spliced in human that was used as comparative reference genome. However, the DNA sequence of the exon 3 can be found about 130 bp upstream of the provided *Vicugna pacos* genomic sequence (Pauciullo and Erhardt, 2015). Furthermore, although the genome sequencing has been completed for the wild feral, dromedary, and Bactrian camels, as well as for the alpaca (Wang et al., 2012; Wu et al., 2014; Fitak et al., 2016), their annotation is still incomplete. Therefore, it is necessary to gain more data to help the annotation in camelids.

In this context, we focused our investigation on genes encoding the main component of milk proteins, providing for the first time a detailed comparative analysis of the casein cluster in camelids; information on haplotype variability in two dromedary populations; and the physical map of the casein genes in alpaca.

### Multiple Bioinformatics Analysis

#### Cluster Organization

Milk proteins and the corresponding coding genes have been deeply studied because they represent in all species the primary source of nutrients for the new born. Caseins (뇸αs1, β, αs2, and κ) are the main component of milk proteins, and they are coded by single autosomal genes (*CSN1S1*, *CSN2*, *CSN1S2*, and *CSN3*, respectively) clustered in a DNA stretch closely linked.

In camelids, the entire casein cluster covers a region slightly less than 190 kb (**Table 2**), and it appears to be “contracted” compared with the cluster observed in other species, in general spread between 250 and 350 kb, depending on the species (Martin et al., 2013; Ryskaliyeva et al., 2018). For instance, the human casein gene cluster is characterized by 11 genes, which exist approximately in the same position in other species. Conversely, the same DNA region in camelids only includes five genes (**Figure 1**).

It is known that the genome expansion is a key mechanism of diversification in the evolution, with gene duplication, exon duplication, and alternative splicing acting as the major driving forces. Jones et al. (1985) proposed that the casein genes evolved from an ancestral gene by a combination of intra- and inter-genic exon duplications. In this respect, some mammals, including horse, donkey, rodents, and rabbit, show two αs2-casein encoding genes (*CSN1S2*a and *CSN1S2*b), which may have arisen by a relatively recent gene-duplication event and may represent examples of paralog duplication (Stewart et al., 1987; Ginger et al., 1999; Cosenza et al., 2010). Conversely, the analysis of the sequences in the intergenic region *CSN2* - *CSN3* of camelids did not evidence the existence of a second *CSN1S2* gene (**Figure 1**). This finding confirms the phylogenetic data of Rijnkels (2002), which demonstrated the gene loss in the *Artiodactyla*, while further divergence between the two gene copies in the other species was partially achieved by differential exon usage. Conversely, the *ODAM* gene was found in camelids and, similarly to the casein genes, it is always present in all species (**Figure 1**).

The organization and the orientation of the casein genes in camelids were conserved as in the other species studied to date (Fujiwara et al., 1997; Rijnkels et al., 1997; Milenkovic et al., 2002; Pauloin et al., 2002; Rijnkels, 2002; Ramunno et al., 2004) (**Figure 1**). Also in camelids, the conservation in the orthologues casein genes is mainly in the 5’ UTR and the signal peptide. In fact, in all casein genes, the first exon encodes the 5’UTR. In the Ca-sensitive caseins (*CSN1S1, CSN2, CSN1S2*), the second exon carries the remaining 12 nucleotides of the 5’UTR and encodes the signal peptide and two amino acids of the mature protein. In the *CSN3* gene, the signal sequence is encoded by the exon 2 and part of exon 3.

The comparative analysis of the genome sequences with the casein transcripts in llamas (Pauciullo and Erhardt, 2015) and dromedary (Kappeler et al., 1998) and the sequences of dromedary *CSN2* (EMBL acc. No. HG969421) and *CSN3* (EMBL acc. no. HE863813) genes allowed the identification of the exons. The architecture of the four genes is extremely “fragmented” in terms of coding regions. The dromedary *CSN1S1* consists of 20 exons, whereas the other camelids shared an organization in 21 exons. The main difference is due to the exon 20, taking as reference the llama cDNA reported by Pauciullo and Erhardt (2015). This exon (44 bp long), partially coding for the termination stop codon (exon 19 5’-TG … A-3’ exon 20), was not found by Kappeler et al. (1998). The reason lies in the mutation that occurred at the donor splice site of dromedary sequence (**Supplementary Figure 1**), which alters the correct identification of the splicing sites and skips out the exon. The correct reading frame is then restored by the next exon, which starts also with an adenine, thus restoring the termination stop codon. Conversely, in the other camelids, the exon 20 and the corresponding splicing sites are conserved. In addition, the analysis of its sequence showed an identity of 95% with the exon 18 of the *CSN1S1* gene (EMBL acc. no. EU025875.1) and cDNA in pig (NM_001004029), and a similarity of 91% with bovine (X59856), goat (AJ504710), and sheep (JN560175) homologous exon.

The *CSN2* gene is conserved in the structure (nine exons) and in the inverted orientation, also in comparison with other species (**Figure 1**).

The *CSN1S2* is arranged in 17 exons in all camelids. This structure confirms the data already published for llamas (Pauciullo and Erhardt, 2015), but it is slightly different from the information reported previously for dromedary (Kappeler et al., 1998). These authors based their study on a reverse approach, from protein to mRNA. The clone library was screened by degenerated primers, whose sequences were deduced from the sequencing of tryptic peptide digestions. Furthermore, they never mentioned the number of clones analyzed; therefore, it is likely that not all mRNA populations were found. To date, no other studies on casein transcripts were carried out in dromedary, but the comparison of the *CSN1S2* genome sequences with the corresponding llama cDNA (Pauciullo and Erhardt, 2015) evidenced that the exon 12 is conserved also in the other camelids, and no mutations affect splicing elements. This exon that is 27 bp long and coding for a peptide of nine amino acids (ENSKKTVDT) is homologous (96.3%) to the predicted αs2 cDNA of *Pantholops hodgsonii* (XM_005985429), a wild Tibetan antelope well adapted to survive in severe conditions, which remind analogous situation of camelids. The same exon shared an identity of 90% with the exon 13 of the bovine (M94327.1) and goat (AJ297316.1) *CSN1S2* gene; about 89% with buffalo (FM865619), sheep (GU169085), and horse (NM_001170767) homologous cDNA; and 85% with the exon 14 of donkey (FN298386.2) *CSN1S2* I gene. Therefore, we postulate that the exon 12 was not described in dromedary *CSN1S2* gene because it was likely spliced out in the pool of clones as analyzed by Kappeler et al. (1998). Recently, Ryskaliyeva et al. (2018) reported a deep characterization of milk protein in Old World camelids accomplished by LC-ESI-MS. However, these authors reported a different phosphorylation level of αs2-CN and did not analyze deeply the primary transcripts. More recently, an extensive protein characterization proposed by the same authors confirmed the splicing of this nine amino acids in the Old World camelids and evidenced new αs2-CN isoforms (Ryskaliyeva et al., 2019). Considering these recent findings, a deep investigation at transcript level is highly beneficial to elucidate all constitutive and alternative splicing events in mRNA maturation process of *CSN1S2*.

Also, the structure of the *CSN3* is different between the Old and New World camelids. The gene arrangement in five exons is very well conserved among the species (Rijnkels, 2002); however, in llamas, 66.6% of the *CSN3* gene transcripts showed one additional “cryptic” exon of 43 bp (Pauciullo and Erhardt, 2015). This extra exon was not identified in the dromedary *CSN3* transcripts (Kappeler et al., 1998), although the sequence is present in the corresponding intron of all camelids *CSN3* gene sequences. Although many nucleotide differences discriminate the intron of both Old and New World camelids, the computational analysis of spliceosome specific sites confirmed the occurrence of the splicing elements: a branch point, a polypyrimidine tract, and a terminal AG acceptor site (score 0.87) at the extreme 3’ end of the intron 1. Moreover, the occurrence of a donor site at the 5’ end of the intron 2 was estimated with a score of 0.99 confirming the existence of the additional exon (**Supplementary Figure 2**).

The occurrence of cryptic exons in the casein genes was already observed in camelids. For instance, in the *CSN1S1* gene of dromedary and llamas, the out-splicing of the exon 18 generates two variants (A and B) differing for the octapeptide (EQAYFHLE). Additional examples exist also in other species. For example, the exon 3 of the human β-casein was described as cryptic due to the interruption of the polypyrimidic tract of the intron 2 by four purines (Menon et al., 1992). In camelids, the occurrence of a larger polypyrimidine DNA tract and the existence of different branch points (BPs) besides the conventional mammalian BP sequence (5’-YTRAY-3’) might be the reason of an alternative skipping of the cryptic exon, as already observed in llamas (Pauciullo and Erhardt, 2015).

The presence of an extra exon in *CSN3* cDNA would also add a new reading frame, *de facto* extending the length of the signal peptide of six amino acids (*MLLGAI*) at NH_2_-terminus (three coming from the cryptic exon 2 and three coming from the reading frame of the following exon). In addition, two possible translation start codon (ATG) would occur (one on the cryptic exon 2 and one canonical on the exon 3), without altering the normal reading frame. Therefore, both protein variants would have a “functional” signal peptide to guarantee the crucial role of κ-CN in the casein micelle maintenance (**Supplementary Figure 2**).

Despite the new information provided by our analysis, a deep investigation at transcript level is needed, at least in dairy camels (dromedary and Bactrian), to elucidate the existence of a real difference in the *CSN3* gene structure between Old (five exons) and New World (six exons) camelids and to clarify the other issues stressed in the present study.

##### Single Nucleotide Polymorphisms

The comparative analysis of the casein cluster among the four investigated camelids showed a high level of genetic diversity. Considering only simple events (nucleotide substitution, insertions, and deletions), a total of 12,818 SNPs were found (**Table 3**).

It is known that genetic polymorphisms contribute to variations in phenotypes. In ruminants, the *CSN1S1* can be surely considered as the most polymorphic gene among caseins (Ramunno et al., 2005; Caroli et al., 2006; Caroli et al., 2009). Although few studies were carried out in camelids, our data confirm the highest level of variation in *CSN1S1* gene (99 SNPs in Bactrian *vs.* 248 in dromedary *vs.* 626 in alpaca), with a total of 24 SNPs occurring in the exons, 13 of which falling in translated regions and, therefore, responsible of amino acids variations among the species.

Currently, at least four protein variants (A, B, C, and D) were detected in the dromedary αs1-CN, and the molecular event responsible for these phenotype variations was clarified in three out of four cases. The alternative out-splicing of the exon 18 differentiates the variant A (207 aa) and B (215 aa). This genetic event is due to the insertion of 11 bp (ATTGAATAAAA) in the intron 17, which negatively affects the secondary structure of the *CSN1S1* A pre-mRNA for the creation of a hairpin and coiled loop (Erhardt et al., 2016). Conversely, the allele C is due to a single nucleotide polymorphism (c.150G > T; GenBank ID JF429138) occurring at the exon 5 and resulting in the amino acid substitution p.30Glu > Asp in the mature protein (Shuiep et al., 2013). Recently, Erhardt et al. (2016) reported a new variant, named D. This showed a different IEF profile, but the analysis of the gene sequence did not evidence any substantial difference with the A allele, apart from an insertion of 11bp in the intron 17. Therefore, the molecular event is still unknown. The *CSN1S1* gene is also polymorphic in llamas, where four protein variants corresponding to four haplotypes were recently reported by Pauciullo et al. (2017). Also in this case, the molecular bases of the differences were identified in two SNPs (c.366G > A, exon 12 and c.690C > T, exon 19) responsible for the amino acid substitutions p.86Val > Ile and p.194His > Tyr, respectively.

The comparison of *CSN2* genes showed 676 polymorphic sites. This gene was the second most polymorphic in camelids with 35 mutations realized in the coding regions, of which 16 occurring in translated regions. The *CSN2* was well characterized in ruminants (Caroli et al., 2006; Cosenza et al., 2007; Caroli et al., 2009), and a detailed description of the gene was also reported in dromedary and Bactrian camels (Pauciullo et al., 2014). To date, only one polymorphism affecting the protein was reported in Bactrians, that is the SNP c.666G > A, which is responsible for the amino acid change p.201Met > Ile (Pauciullo et al., 2014), recently confirmed by Ryskaliyeva et al. (2018). SNP discovery in dromedary highlighted two single polymorphisms: the SNP g.4175C > A that occurred within the codon 7 of the signal peptide, but it was a synonymous mutation (GCC^Ala^ > GCA^Ala^); and the SNP g.2126A > G that occurred in TATA-box of dromedary *CSN2* promoter and was more interesting because it putatively affects the transcription factor binding activity (Pauciullo et al., 2014).

A similar number of genetic markers (644) were also found by the comparative analysis of the *CSN1S2* genes. This gene resulted the least polymorphic in dromedary with 79 SNPs in total (**Table 3**). The SNPs affecting the exons were 27, of which 17 occurred in translated regions and, therefore, putatively responsible of amino acids differences. As for the other casein fraction, also the *CSN1S2* was well studied in ruminants, and many alleles were found (Caroli et al., 2006; Cosenza et al., 2007; Caroli et al., 2009). Exons skipping are also considered as frequent events for the αs2-casein encoding gene in different species (Boisnard et al., 1991; Bouniol et al., 1993; Cosenza et al., 2009). However, to date, no genetic variants or alternative transcripts have been reported in camelids. Considering the origin and the structure of the *CSN1S2* gene (Rijnkels, 2002), at least rearrangements resulting from alternative splicing of mRNA are expected. Therefore, surely in camelids, this casein gene deserves more attention at gene transcript level.

The least polymorphic gene in camelids was the *CSN3,* with 569 markers found by the comparative analysis (**Table 3**). Twenty-one SNPs were found in the exons, and 14 of them occurred in the translated regions, mostly located in the exon 4 (12 SNP found only in Alpaca). The *CSN3* is not evolutionarily related to the Ca-sensitive casein genes, but is physically linked to this gene family, and is functionally important for stabilizing the Ca-sensitive caseins in the micelle. Therefore, mutations occurring in this gene can be particularly important for the biological role carried out by the κ-CN. Pauciullo et al. (2013a)carried out genetic diversity discovery in Sudanese dromedary. However, no polymorphisms were found in the coding regions, whereas the only interesting SNP was found in the promoter region (g.1029T > C) because affecting the consensus site for the transcription factor HNF-1 just upstreams the exon 1. So far, many genetic variants of κ-casein were identified at protein or DNA level in many species. The absence of polymorphisms in *CSN3* coding regions suggests that the level of genetic variations in camel κ-casein is very low in comparison with other species (Caroli et al., 2006; Carneiro and Ferrand, 2007; Hobor et al., 2008; Caroli et al., 2009). Therefore, a deeper analysis of camel *CSN3* would be necessary to search for genetic diversity, and further studies would be required in order to assess the potential impact on the quali-quantitative properties of camel milk. For example, it is known that dairy cows with the genotype *CSN3* BB produce milk with a significantly higher protein content (Caroli et al., 2009). This led the dairy farmers to select preferentially these cows in order to have a higher cheese yield. Dairy camel breeders could exploit similar advantages, because the presence of quantitative alleles cannot be excluded also in camels. Moreover, Weimann et al. (2009) showed that in cattle *CSN3*, variants are source of different angiotensin I converting enzyme (ACE) inhibitor peptides and revealed their potential role for human health. These bio-functional peptides were found also in camel milk (Al hay and Al Kanhal, 2010). Therefore, it is likely that the genetic variants of the camel κ-casein might also influence its functional role, giving the camel milk an additional value for the human nutrition.

##### Microsatellites

Despite the progress of genomics and the availability of the high-throughput genotyping platforms, for many domestic species, including camelids, microsatellite analysis still represents a powerful tool for the genetic identification and assessment of parentage analysis in camelids (Penedo et al., 1999; Evdotchenko et al., 2003; Mburu et al., 2003) and characterization of the domestication process of the dromedary (Almathen et al., 2016). The analysis of sequences for microsatellites discovery showed 35 short tandem repeats (**Table 4**). A high level of polymorphism was found among the species (72.7%), demonstrated by 17 microsatellites showing a different number of repeats and seven species-specific short tandem repeats (one in Bactrian, two in dromedary, and four in alpaca), since no over-lapping sequences were found. Although no information is available on allelic diversity, the latter microsatellites can potentially become very useful for species discrimination, genetic diversity, and population structure studies or, simply, for parentage assignment, which is a service in high demand for the camel racing industry (Penedo et al., 1999; Spencer et al., 2010). Furthermore, such a panel of markers, together with other microsatellites distributed along the same chromosome (or SNPs that provide complementary information), gives the opportunity to begin the search for QTLs of economic importance.

#### Interspersed Elements

Transposable elements have played a fundamental role in species diversification, influencing the evolution of mammalian genomes (Bowen and Jordan, 2002). Camel genome contains about 34% of repetitive DNA (Wang et al., 2012; Fitak et al., 2016), mainly belonging to SINEs and LINEs expanded in the genome by a process known as retroposition. Compared to the whole genome, the DNA fragment containing the casein cluster showed a lower level of repetitive DNA (on average 19.8%). However, the transposition process probably happened in a widespread coverage, within and outside the casein genes, as demonstrated by the short distance occurring between the interspersed elements in each of the investigated species (**Supplementary Table 1**). The comparative analysis showed that 94.4% of the repetitive DNA was shared between two or more species of camelids, whereas 39 interspersed elements (5.6%) were species specific (**Table 5**).

These interspersed elements are useful for a better understanding of the divergent evolution of camelids within the *Tylopoda* family. Recently, the genome analysis of camelids elucidated the divergent time of the ancestors of the New and Old World camelids, indicating that the division between Camelini and Lamini occurred in North America about 16.3 Mya (Wu et al., 2014). Considering this divergence time, it is evident that the interspersed elements common to all camelids were already present in the ancestor genome, whereas the repetitive elements typical of each species were introduced after the separation of Camelini and Lamini tribes. Alpaca showed 27 species specific transpositions, whereas only 12 in the Old word camelids (four in wild feral, three in Bactrian, and five in dromedary). Considering that transposition insertions reflect the level of genome size expansion (Liu et al., 2003), the alpaca genome probably underwent to a more intensive extension due to lineage-specific shifts in transposition activity within the last 17 million years of evolution. This is confirmed by the larger size of alpaca genome (2.05 Gb) compared to that of the Bactrian (2.01 Gb) and dromedary (2.01 Gb) (Wu et al., 2014). Since transpositions are considered powerful mutagens at gene level, their impact on phenotypic change and evolution of camelids might be more significant than considered so far. Examples of phenotypic changes for transposition insertions are present also in the casein genes that, also in this respect, represent a very useful model of study. For instance, the allele E of the *CSN1S1* in goats is characterized by the insertion of a truncated LINE of 457 bp in the last exon, which is responsible of a three-fold reduction of transcriptional rate of the corresponding protein (Pérez et al., 1994). Similarly, in cattle, the *CSN1S1* allele G showed a truncated LINE of 371 bp at the exon 19. The interaction between the LINE sequence and the poly(A) sequence of the mature transcript, reduced the mRNA stability causing a rapid degradation of the transcript and a limited protein synthesis efficiency (Rando et al., 1998).

The presence of repetitive DNA within casein genes in dromedary was already evidenced in *CSN2* (Pauciullo et al., 2014) and *CSN3* genes (Pauciullo et al., 2013b). In these studies, LINEs belonging to L1MA family were found to be species specific in comparison to cattle. None of them affected the exon structure; therefore, no influence on mRNA is expected, as well as on protein production. Furthermore, a lower number of repetitive elements were found in dromedary compared to cattle, thus indicating that *Tylopoda* diverged from Ruminantia before additional transpositions occurred at different times during the divergence of such suborder (Nijman et al., 2002).

#### Promoters

Five hundred five motifs for transcription factors enhancing and/or repressing the casein gene expression were found. For brevity, **Table 6** reports only the motifs shared by the four casein promoters and showing higher binding scores. The consensus sequences belonging to the octamer-binding family (Oct), GATA-binding proteins, C/EBPs (CCAAT-enhancer-binding proteins), and ubiquitous activators like Sp1, Ap1, and Ap2, were found more frequently because they are closely linked to protein and milk production.

In particular, 33 C/EBP motifs, 11 mammary gland factor/STAT5 (MGF/STAT5), and 63 octamer-binding protein (Oct-1) were found. These elements initiate the transcription through sinergyc interactions with other motifs (Wyszomierski and Rosen, 2001). For instance, Oct-1 and STAT5 are considered as co-activators, and they can stimulate casein gene expression by hormonal induction (Zhao et al., 2002). In addition, Oct-1 can affect acute myeloid leukemia (AML) factors by reducing its inhibitory role in the DNA binding and creating a complex that stimulates the expression of casein genes (Inman et al., 2005).

The activation of casein expression can be mediated also by hepatocyte nuclear factors-3 (HNF3) by a combined action with nearby C/EBP and glucocorticoid elements (GR) (Schild and Geldermann, 1996; Christoffels et al., 1998). Analogous interactions are supposed for the MyoD transcription factor (Jiang and Zacksenhaus, 2002) and for Pbx1 in a synergic action with glucocorticoid receptors (Subramaniam et al., 2003). Many other motifs were found, including Sp1, NF, YY1, etc., as it was already described in previous studies (Kappeler et al., 2003; Pauciullo et al., 2013a; Pauciullo et al., 2014). However, it is remarkable to point out the existence of one SREBP (sterol regulatory element-binding protein) at position (-61/-51) of the *CSN3* promoter. Although the most known function of this transcription factor is the regulation of genes involved in milk fat pathway (Harvatine and Bauman, 2006), Reed et al. (2008) reported also a down regulation role of SREBP in the expression of caseins.

The description of the most occurring motifs regulating the casein gene expression opens the way to functional studies, which will be necessary to evaluate the influence of these elements on the transcriptional regulation of casein genes in camelids.

### Experimental Data

#### Genotype and Haplotype Analysis

The genotyping of 267 dromedaries for the SNPs at *CSN1S1*, *CSN2*, and *CSN3* showed similarities and differences in the allelic frequencies of the two camel populations. At *CSN1S1*, the variant C (c.150T, p.30Asp) had a very low frequency (< 0.1) in both populations, even lower than that reported by Shuiep et al. (2013) (mean frequency of the allele C = 0.158). Furthermore, this variant does not characterize the other camelids, all carrying the guanine (c.150G, p.30Glu) that can be considered as the ancestral condition within the *Tylopoda* family.

The allele C induces the amino acid replacement p.30Glu > Asp evidenced at protein level by IEF and confirmed at DNA level by the SNP c.150G > T (Shuiep et al., 2013). Taking as reference the variant A of the *CSN1S1*, we carried out bioinformatics analysis to predict the effect of the amino acid change in the secondary structure of the protein and to assess whether it could have an impact on its biological function. The analysis showed an evident change in the secondary structure of α-helix that partially turned to β-sheets (**Supplementary Figure 3**). Furthermore, this structural change in the complex affected a wider region of the protein (amino acids 20–50). However, despite the structural change, PROVEAN analysis showed a score of 0.778, which classifies the mutation as neutral. It is known that any modification of the secondary structure of a protein likely means a change also in the final protein form. If this happens, the functionality of the protein may be affected. This is extremely important in a protein complex such as casein micelle, where the Ca-sensible caseins (αs1-; β and αs2-CN) are closely linked and grouped together in a balanced condition kept by the κ-CN. Examples of strong and defective alleles due to “simple” amino acid changes are known in goats (Cosenza et al., 2008), cattle (Caroli et al., 2009), sheep (Giambra et al., 2010), buffalo (Cosenza et al., 2009), etc. Therefore, further studies are necessary to assess the impact of this variant on the micelle stability, as well as on technological properties and nutrition aspects of the dromedary milk and the related dairy products.

A different situation was observed for the other two SNPs analyzed (g.2126A > G, *CSN2*, and g.1029T > C, *CSN3*), which showed inverted allele frequencies in the investigated populations (**Table 7**). In our knowledge, no genetic programs or selection strategies are applied on camels in both countries (Sudan and Nigeria); therefore, such a difference might be indicative of other effects like genetic drift and/or inbreeding. Nowadays, camel population in Nigeria numbers about 300,000 heads (in 1961, they were only 14,000) and no intensive importing flow of live camels (only 1,300 heads) is documented in the years 1961–2016 (www.faostat.org). Therefore, the current allele distribution could be generated by a founder effect during their domestication time, and the lack of gene flow might have played a role in the differentiation of the Nigerian from the much widespread Sudanese population. This assumption should be confirmed by genetic comparisons with other dromedary populations. However, Nigerian dromedaries were investigated using microsatellites and mitochondrial DNA analysis, and genetic diversity has been found in comparison with Australian, Kenyan, and Canarian Islands populations, assuming inbreeding and/or founder effects as possible reasons (Abdussamad et al., 2015).

On the basis of genotypes detected at each *locus*, eight haplotypes were observed in both populations and, overall, three of them (GAC, GAT, GGT) accounted for more than 80% of the observed variability, with the haplotype GAC most represented (0.288). The haplotype TGC was the rarest observed (0.005), and additional three had very low frequency (from 0.011 to 0.042). Sudanese camels showed a higher frequency of the haplotype GAC (0.348) compared to the Nigerian (0.187), where the most represented haplotype was GGC (0.290), rather underrepresented in the Sudanese camels (0.028). Ecotypes within Sudanese population showed further differences. For instance, Shanbali and Lahaoi *vs.* Khali and Arabi showed nearly opposite frequencies for the haplotypes GAC and GAT, thus potentially opening the possibility for a rapid directional selection if future studies will demonstrate associations with milk properties.

The knowledge of haplotypes is particular useful in breeding schemes because they may impact on a trait in a different way compared to single alleles, exploiting all the genetic effects existing among individual genes. This would be particularly convenient for the casein genes, which are closely linked. Therefore, a deeper screening of casein variability should be accomplished in dairy camels at both protein and DNA level to have a better knowledge on the amount and potential use of the genetic polymorphisms at these loci.

#### Cytogenetic Mapping

Casein genes are mapped on the same chromosome in all species investigated so far. For instance, they are located on chromosome 6 in cattle, sheep and goat, on chromosome 4 in humans, 8 in pig, 14 in rat, 3 in horse, etc. (Rijnkels, 2002, Martin et al., 2013). Conversely, the cytogenetic map of the casein genes was never reported in camelids and, in general, very little information is available so far on the physical mapping of other *loci* (Avila et al., 2014a; Avila et al., 2014b; Perelman et al., 2018).

The production of specific probes allowed mapping the casein genes to the chromosome 2q21. Such result also confirms the comparative evolutionary study of Balmus et al. (2007). In fact, cross-hybridization experiments with molecular painting probes evidenced that the dromedary camel chromosome 2 (CDR2) corresponds to the bovine chromosome 6 (BTA6) where the casein genes have been mapped (Rijnkels, 2002). Furthermore, the extensive similarities reported in the karyotypes of the camelids (*Camelus dromedarius*, *Camelus bactrianus*, *Lama glama*, *Lama guanicoe*, *V. pacos* and *Vicugna vicugna*) (Bunch et al., 1985; Di Berardino et al., 2006; Balmus et al., 2007) confirm that CDR2 and VPA2 are homologous chromosomes of related species.

This result is also interesting for its potential to physically map other genes on camel chromosome 2. For instance, the spotting *locus* responsible of white-spotting phenotypes in cattle was mapped on BTA6, in a chromosomal region including the *KIT* gene (Grosz and MacNeil, 1999), approximately 15 Mbp upstream the casein cluster. The white-spotting phenotype is an undesired characteristic in alpacas, which are mainly bred for the quality of their coat fibers (extremely fine, hypoallergenic, and naturally stained). Therefore, studies on the genetic variability of the casein cluster in alpacas might be of interest to identify and select alleles in linkage disequilibrium with favorable coat characteristics. On the other site, the so called “blue-eyed white phenotypes” are in some cases associated with congenital deafness (Gauly et al., 2005) and associated with the *KIT* locus in many species, including alpacas (Jackling et al., 2014).

## Conclusion

The knowledge of casein genes in camelids herein summarized provide fundamental information useful for different applications, such as biodiversity analysis or association studies functional characteristics (dietetic, technological, and nutraceutical) of camel milk to better meet the consumers’ requirements.

Nowadays, planning the production of milk with different protein properties suitable for its specific use is a realistic challenge for breeders and an important goal for animal geneticists. In this respect, all the genetic variability found is useful in selection programs of dairy camels for better exploiting the effects of the entire casein cluster on milk yield and its related traits.

## Author Contributions

AP and GE conceived and designed the experiments. AP performed the experiments. AP and GC analyzed the data. GE and LD contributed reagents/materials/analysis tools. AP wrote the paper. AP, ETS, MDO, GC, LD, and GE revised the article critically for important intellectual content. AP, ETS, MDO, GC, LD, and GE gave final approval of the version to be published.

## Funding

This research was financially supported by the project Camilk (PAUA_CONTR_FIN_18_01), the King Baudouin Foundation United States (KBFUS) Grant number 20180252.

## Conflict of Interest Statement

The authors declare that the research was conducted in the absence of any commercial or financial relationships that could be construed as a potential conflict of interest.
