# Supplementary material for: Casein Gene Cluster in Camelids: Comparative Genome Analysis and New Findings on Haplotype Variability and Physical Mapping
Source: Front Genet. 2019 Aug 29;10:748. doi: 10.3389/fgene.2019.00748 (PMC6726744; doi:10.3389/fgene.2019.00748)
Supplement: Supplementary file 1 [file Table_1.docx]

Intron 19 Exon 20 – 3’UTR

**AS**

#Feral cattacctac gtcacgtgat tttaacaatt tttttttcct tcag**A**AGAGT TAAGTGAATT CTCAGGAACT CCACAATTAT

#Bactr .......... .......... .......... .......... .......... .......... .......... ..........

#Drome .......... .......... .......... .......... .......... .......... .......... ..........

#Alpac g......... ....t..... .......g.. .......... .......... .......... .......... .........c

**DS**

Intron 20

#Feral GGCCTTTG**g**t aagttggaaa tcacttgtct aaccattgat tctcttttca tgtgaggact cagtacaaag atacatgact

#Bactr .......... .......... .......... .......... .......... .......... .......... ..........

#Drome ........**t**. .......... .......... .......... .......... .......... .......... ..........

#Alpac .......... .......... .......... .......... .......... .......... .......... ...t......

Supplementary figure 1. Alignment of the DNA region spanning from the intron 19 to intron 20 of the *CSN1S1* gene in camelids. Dots correspond to identical nucleotide of the upper line (wild feral sequence). The exon 20 is indicated in brackets, acceptor and donor splicing sites are in boxes. In red, the polymorphism occurred at the donor site of the dromedary *CSN1S1*.
