# Supplementary material for: Casein Gene Cluster in Camelids: Comparative Genome Analysis and New Findings on Haplotype Variability and Physical Mapping
Source: Front Genet. 2019 Aug 29;10:748. doi: 10.3389/fgene.2019.00748 (PMC6726744; doi:10.3389/fgene.2019.00748)
Supplement: Supplementary file 2 [file Table_2.docx]

#Feral AATGTAGGAG GAAGATAAAT CACATGCTGC AAAGCACTAA CACCCTTTAA TTAGTCTCCA GTTATTTACC TTGGGTCTtc

#Bactr .......... .......... .......... .......... .......... .......... .......... ..........

#Drome .......... .......... ......A... .......... .......... .......... .......... ..........

#Alpac .......... .......... ......A... .......... T......... .......... .........T ..........

**DS**

**Exon 1**

#Feral tttacagttg agaggccagc tcaacctact gccaagcaag acctgacagg cacaagggaa g**GT**AATCAAA TTGAAATGTC

#Bactr .......... .......... .......... .......... .......... .......... .......... ..........

#Drome ....T..... .......... .......... .......... .......... .......... .......... ..........

#Alpac .......... ........A. .......... .......... .......G.. .......... ........T. ..........

#Feral CAGAGAGAAT GACTCTTTTT CACAGGAGTT AATCATAATT TCTTCCTCTT AGTTTTTATT GCTAAGCTTG AATATGCCTT

#Bactr .......... .......... .......... .......... .......... .......... .......... ..........

#Drome .......... ....T..... .......... .......... .......... .......... .......... ..........

#Alpac .......... .......... .......... .G........ .......... ..C....... .......... ..........

#Feral GGCATCATTT TGACATTAGT TTCTAACCTA AACCTTAGAT TCTGTATAAT GTTATGATTA AACTTATTTT TAACTTCACT

#Bactr .......... .......... .......... .......... .......... .......... ..T....... ..........

#Drome .......... .......... .......... .......... .......... .......... ..T....... ..........

#Alpac .......... .......... .......... .......... .......... .......... ..T....... ..........

#Feral TTGGGTTATT TTTGTCTTCA CTACTGGCTA AACTACTGAA GACAATGTAA ATTGTAAAGA AAAGTTGTTC AAGACAATGA

#Bactr .......... ...A...... .......... .......... .......... .......... .......... ..........

#Drome .......... .......... .......... .......... .......... .......... .......... ..........

#Alpac .......... ...A...... ........C. .......... .......... .......... .......... ..........

#Feral ATTTATTCCT ACAGACATAT GTGGAGGGCT AACTTCTCAG AAAGATGAAT GTGTCATAAT AATCCAGACT GACCCCTTAA

#Bactr .C........ .......... .......... .......... .......... .......... ........T. ..........

#Drome .C........ .......... .......... .......... .......... .......... ........T. ..........

#Alpac .C........ .......A.. .......... .......... .......... .......... ........T. ..........

#Feral CTAATTTAAA ATTTTTATTT CTATCTAAAG ATTATAATTT TATACGTTAC GGTATAACTT TTTTAAAAAT TATGAGTTAC

#Bactr .......... .......... .......... .......... .......... .......... .......... ..........

#Drome .......... .......... .......... .......... ....T..... .......... .......... ..........

#Alpac T......... .......... ...G...... .......... ...TT....T A......... ...A...... ..........

#Feral ATATCCTCAT GAAAGCTGAA TAGTTCTGCT GTTTCATAGA CTTTTCCATC AAGTACTCAT TGGTTTAAGT AACATAAGTA

#Bactr .......... .......... .......... .......... .......... .......... .......... ..........

#Drome .........C .......... .......... .......... .......... .......... .......... ..........

#Alpac T..C.....C .......... .......... .......... .......... ........G. .......... ..........

#Feral AATTATGGTC TTATCTCTCT GATTTCTGTT TATGCTAAAA AAGAGGATCT ACTGTCATTT TACAGATTGC TTGTGTG--A

#Bactr .......... .......... .......... .......... .......... .......... .......... .......--.

#Drome .......... .......... .......... .......... .......... .......... .......... .......--.

#Alpac ..G....... .......... .......... .......... ....A..A.. ...A...... .......... .......TG.

#Feral CTGTGTGTGT AAATCTGTAT ATGTGTTATA ATTTTGCTTG AATTGAAAAC ATCCTAAGAG AGATCTTAAC TATTCCAACT

#Bactr .......... .......... .......... .......... .......... .......... .......... ..........

#Drome .......... .......... .......... .......... .......... .......... .......... ..........

#Alpac .......... .......... .......... ...C...... .......... ..T....... .......... ........T.

#Feral ACAAAAAAGA AATGATAATT ATGTGACATG ATAGAAGTGG TAATTATCAC TACAATGGCA ATCACATTAC AATATATAAA

#Bactr .......... .......... .......... .......... .......... .......... .......... ..........

#Drome .......... .......... .......... .......... .......... .......... .......... ..........

#Alpac .......... .......... --........ .......... .......... .......... .......... ..........

#Feral TATATCAAAT CAACATGTCA TATAATTTAA ATTTACAGAA TGTTATATGT CTAATATATT TGTGTTAAAA AATGAAAAAA

#Bactr .......... .......... .......... .......C.. .......... .......... .......... ..........

#Drome .......... .......... .......... .......C.. .......... .......... .......... ..........

#Alpac .......... .......... ....G..... .......C.. .......... .......... .......... ..........

#Feral GGTAACTGGC AAGTGAAGAA TTAATAGGGA ACTCCATTCT TTTCATTGAA ATTTGAGTTT GCTTGTAGAT CTCACATGAG

#Bactr .......... .......... .......... .......... .......... .......... .......... ..........

#Drome .......... .......... .......... .......... .......... .......... .......... ..........

#Alpac .......... .......... .......... .......... .......... .C........ .......... ..........

#Feral CTCTTTTCCA ACCAATGATA TCCTATCAGA GGTTCTGAAA TCAGTTTAGT AGACAGTCTT TCTAATCATT ATTATCTGTG

#Bactr T......... .......... .......... .......... .......... .......... .......... ..........

#Drome T......... .......... .......... .......... .......... .......... .......... ..........

#Alpac T......... .......... ...C...... .......... ....C..... ...T...... .......... ..........

#Feral AATCAAAAGC AAATGGAGTC TGGTGTGTTC CCTTTACCAA GCCTGGACTT TAACTCTGTG ACCCTCTCTT GTAAAACTCA

#Bactr .......... .......... .......... .......... .......... .......... .......... ..........

#Drome .......... .......... .A........ .......... .......... .......... .......... ..........

#Alpac .......... .......... .......... .......... .......... .......... .......... ..........

#Feral GTTTGCAAGT CTCAAATCTA TACAAGTAGG TCAATAACAG TAGTTGCAAC AAGATGTGGT TAGCATTTAT TTCACAAATA

#Bactr .......... .......... .......... .......... .......... .......... .......... ..........

#Drome .......... .......... .......... .......... .......... .......... .......... ..........

#Alpac ........A. .......... .......... .......... ...C...... .......... .......... ..........

#Feral CCACATATAC TGAAATATTA AAAGCTTAAA CTGTTATATG TATCTTTCTA AAAAGACTAT TTTTAAAAAA TCAAATAAGC

#Bactr .......... .......... .......... .......... .......... .......... .......... ..........

#Drome .......... .......... .......... .......... .......... .......... .......... ..........

#Alpac ...T...... .......... .......... .......... .......... .......... .......... ..........

#Feral AGGTGTGACT CCTATACTCC TTTAGAAAGA TAAGGCTAGT TTATTTCTTT AAATATTTCA GTCAAAGCCC TGTCATTGTC

#Bactr .......... .......... .......... .......... .......... .......... .......... ..........

#Drome .......... T......... .......... .......... .......... .......... .......... ..........

#Alpac .T........ .......... .......... ........A. .......... .......... .......... ....G.....

#Feral ATACCTATGT TTTTATTAAA ACAATCAAGT TAATCTCATC ATTCATGATG ACTGGAGAAG TAATTGTGAA GAATGCAGTT

#Bactr .......... .......... .......... .......... .......... .......... .......... ..........

#Drome .......... .......... .......... .......... .......... ......A... .......... ..........

#Alpac .......... .......... .......... .......... ....G..... .......... .......... ..........

#Feral TCAGTATACC CTCCATCTCC TCCATCTTAC ATGTTCCTGT TAGACCAATC ATGGAATTAC AGAATTTTAG AAATTTTAGT

#Bactr .......... .......... .......... .......... .......... .......... .......... ..........

#Drome .......... .......... .......... .......... .......... .......... .......... ..........

#Alpac .......... .......... .......... .......... .......... .......... .......... ..........

#Feral TCAAACTTTC ATTTATAGGT AAGAATAGTA AGGCCCAGAT AAAGGAAATT ACTTGGACAA AATTGCACAA GTAGAGATAG

#Bactr .......... .......... .......... .......... .......... .......... .......... ..........

#Drome .......... .......... .......... .......... .......... .......... .......... ..........

#Alpac .......... .......... .......... .......... .......... ......G... ......T... ..........

#Feral AGCCTGAGAT GGAACTCAGT TCTCCAAAGG AATGTGTAGT CACACAGTCT CTGGTCCTGA ACCACTCAGC TGGTTTCCCT

#Bactr .......... .......... .......... .......... .......... .......... .......... ..........

#Drome .......... .......... .......... .......... .......... .......... .......... ..........

#Alpac .......... .......... .......... .......... .......... .......... .......... ..........

#Feral TGTTTACAAA GCCTTGCCTA -CTCTTCAGA CTGGCATGGG AAGCCTCTCC TTTTGCTGCC CTAAACCTTC TTTCCAACTT

#Bactr .......... .......... -......... .......... .......... .......... .......... ..........

#Drome .......... .......... -......... .......... .......... .......... .......... ..........

#Alpac ....C..... .......... A......... .......... .......... .......... .......... ..........

#Feral TATCTTTCCC TTTTCTCCAT TTAAATGCCC ATGGATATTC AGAAGGCTTT CTCTTCTATT TGAATGTTCT CTATTAATCT

#Bactr .......... .......... .......... .......... .......... .......... .......... ..........

#Drome .......... .......... .......... .......... .......... .......... .......... ..........

#Alpac .......... .......... .....A.... .......... .......... .......... ....G..... ..........

#Feral CTGCATGAGT AAGTCCCACC TGTATCTCCC ACACAAGTAG TTTGTTGATT TCTTAACTGA AAACTTCTAT ATGTAGAAAG

#Bactr .......... .......... .......... .......... .......... .......... .......... ..........

#Drome ....G..... .......... .......... .......... .......... .......... .......... ..........

#Alpac ....G..... .......... .......... .......... .......... .......... .......... ..........

**BP**

**BPs**

**BP**

**AS**

**DS**

**(Py)n**

**Cryptic exon 2**

***M L L***

#Feral CCACTTTTGT GACATTTTCG ACTTTCTTTC CTCTAAC**AG**t gacttgttgt tgcaactctc ttcctacaat tg*ATGCTACT*

#Bactr .......... .......... .......... .......... .......... .......... .......... ..........

#Drome .......... .......... .......... .......... .......... .......... .......... ..........

#Alpac .......... .......... .......... .......... .......... .......... .......... ..........

#Feral GG**GT**AAGACT AGCAAGACCA ATTCACCTTG ATAATCTCAA CACTTGACAG TGCCTT---G TTCTTTATAT ATATAAGCTA

#Bactr .......... .......... .......... .......... .......... ......---. .......... ..........

#Drome .......... .......... .......... .......... .......... ......---. .......... ..........

#Alpac ........ ....... .. . .......... .......... .......... ......CTT. .......... .....T....

#Feral AAATTTTTAT TTATAGGTAA GAATAGTACC TATAAATGTA CATATATATT TATAGGCACC ATTATGTGCC TATACATTAT

#Bactr .......... .......... .......... .......... .......... .......... .......... ..........

#Drome .......... .......... .......... .......... .......... .......... .......... ..........

#Alpac .......... .......... .......... ..G....... .......... .......... .......... ..........

#Feral ATATGTGTAT GTATATGTGT ATACATAGGG ACACATTTTT TTCCAACAAA GTTTAAAGTT ATTCAAACTA CCTGTCCTTC

#Bactr .......... .......... .......... .......... .......... .......... .......... ..........

#Drome .......... .......... .......... .......... .......... .......... .......... ..........

#Alpac .......... A.......A. .......... .......... .......... .......... .......... ..........

#Feral TCTACAACAG AATAAAATTC TCATGTAATT TAATTATTTA TAAAAAATTG TGTCTATGAC TTTCCTTATT AATGGCCTAC

#Bactr .......... .......... .......... .......... .......... .......... .......... ..........

#Drome .......... .......... .T........ .......... .......... .......... .........A ..........

#Alpac .......... .......... .T........ .......... .......... .......... .......... ..........

#Feral TAGTAGGTTC CTGGTACATG TTAAATGAAT ATATGATTAA CCTTGTAATG ATACTTTTAA TATTATAGCC AAAAATCAAA

#Bactr .......... .......... .......... .......... .......... .......... .......... ..........

#Drome .......... .......... .......... .......... .......... .......... .......... .....C....

#Alpac .......... .......... .C........ .......... .......... .......... .......... ....G.....

#Feral TTCATACACA TTATACTGTT TGGTTTTTCT AAAGTTTTTA ATAAAATAAA TTTACCACAT TGGCTATATC TACCCATAAA

#Bactr .......... .......... .......... .......... .......... .......... .......... ..........

#Drome .......... .......... .......... .......... .......... .......... .......... ..........

#Alpac .......... .......... .......... .......... G......... .......... .......... ..........

#Feral TCTAGTGGGG TTTTTCCTTA AAGCAAAGAA AACATTGTTC CAAATCACTC CTAAATTATT AGTAATAGAT CCTTTTAATT

#Bactr .......... .......... .......... .......... .......... .......... .......... ..........

#Drome .......... .......... .......... .......... .......... .......... .......... ..........

#Alpac .......C.. .......... .......... .......... .......... .......... .....C.... ..........

**AS**

**Exon 3**

***G A I M K S F F L V V T I L A***

#Feral TAATTTTTTT TTAATTTTTT AATTTTTATT TTT**AG**GTGCA ATCATGAAGA GCTTCTTCCT AGTTGTGACT ATCCTGGCAT

#Bactr .......... .......... .......... .......... .......... .......... .......... ..........

#Drome .......... .......... .......... .......... .......... .......... .......... ..........

#Alpac ........-- ---------. ...C...... .......... .......... .......... ...C...... ..........

***L T L P F L***

#Feral TAACTCTACC TTTTTTGGTG

#Bactr .......... ..........

#Drome .......... ..........

#Alpac .......G.. ..........

Supplementary Figure 2. Alignment of the DNA fragment of the *CSN3* gene (κ-casein) including the ‘cryptic’ exon. Dots represent identical nucleotides to those in upper lines. The branch point site is fully underlined and the bold adenine represent the main branch point (black triangle), followed by a polypyrimidine tract (Py_n_) and the acceptor splice site (AS, score 0.87). The dotted-line and the white triangles represent additional polypyrimidine tracts and alternative branch points, respectively. The donor splice site (DS, score 0.99) for the following intron splicing is normally present. The signal peptide is in bold italics, the cryptic ATG is waived and the canonical ATG is double waived.
