# Supplementary material for: Casein Gene Cluster in Camelids: Comparative Genome Analysis and New Findings on Haplotype Variability and Physical Mapping
Source: Front Genet. 2019 Aug 29;10:748. doi: 10.3389/fgene.2019.00748 (PMC6726744; doi:10.3389/fgene.2019.00748)
Supplement: Supplementary file 3 [file Table_3.docx]

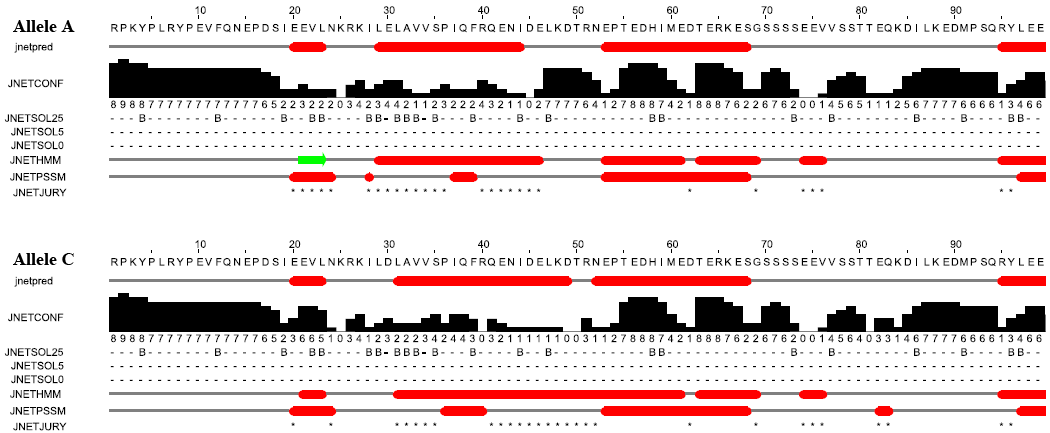


Supplementary figure 3. Comparative analysis of the secondary structure of the αs1-casein for the variant A (p.30E) *vs* variant C (p.30D), predicted by Jpred 4 software. The yellow rectangle denotes the different amino acid, whereas the blue box indicates the protein portion affected by the amino acid replacement. Red tubes indicate the α-helix, the green arrow shows the β-sheet.
